# Association between the plasma-to-red blood cell ratio and survival in geriatric and non-geriatric trauma patients undergoing massive transfusion: a retrospective cohort study

**DOI:** 10.1186/s40560-022-00595-7

**Published:** 2022-01-11

**Authors:** Mitsuaki Kojima, Akira Endo, Atsushi Shiraishi, Tomohisa Shoko, Yasuhiro Otomo, Raul Coimbra

**Affiliations:** 1 Emergency and Critical Care Medicine, Tokyo Women’s Medical University Adachi Medical Center, 4-33-1 Kohoku, Adachi-ku, Tokyo, Japan; 2 Trauma and Acute Critical Care Medical Center, Tokyo Medical and Dental University Hospital, 1-5-45 Yushima, Bunkyo-ku, Tokyo, Japan; 3 Emergency and Trauma Center, Kameda Medical Center, 929 Higashicho, Kamogawa, Chiba Japan; 4 Comparative Effectiveness and Clinical Outcomes Research Center-CECORC, Riverside University Health System Medical Center, 26520 Cactus Ave., Moreno Valley, CA USA

**Keywords:** Damage control resuscitation, Geriatric trauma, Massive transfusion, Trauma registry

## Abstract

**Background:**

The benefits of a high plasma-to-red blood cell (RBC) ratio on the survival of injured patients who receive massive transfusions remain unclear, especially in older patients. We aimed to investigate the interaction of age with the plasma-to-RBC ratio and clinical outcomes of trauma patients.

**Methods:**

In this retrospective study conducted from 2013 to 2016, trauma patients who received massive transfusions were included. Using a generalized additive model (GAM),we assessed how the plasma-to-RBC ratio and age affected the in-hospital mortality rates. The association of the plasma-to-RBC ratio [low (< 0.5), medium (0.5–1.0), and high (≥ 1.0)] with in-hospital mortality and the incidence of adverse events were assessed for the overall cohort and for patients stratified into non-geriatric (16–64 years) and geriatric (≥ 65 years) groups using logistic regression analyses.

**Results:**

In total, 13,894 patients were included. The GAM plot of the plasma-to-RBC ratio for in-hospital mortality demonstrated a downward convex unimodal curve for the entire cohort. The low-transfusion ratio group was associated with increased odds of in-hospital mortality in the non-geriatric cohort [odds ratio 1.38, 95% confidence interval (CI) 1.22–1.56]; no association was observed in the geriatric group (odds ratio 0.84, 95% CI 0.62–1.12). An increase in the transfusion ratio was associated with a higher incidence of adverse events in the non-geriatric and geriatric groups.

**Conclusion:**

The association of the non-geriatric age category and plasma-to-RBC ratio for in-hospital mortality was clearly demonstrated. However, the relationship between the plasma-to-RBC ratio with mortality among geriatric patients remains inconclusive.

**Supplementary Information:**

The online version contains supplementary material available at 10.1186/s40560-022-00595-7.

## Background

Massive transfusion (MT) plays a critical role in the resuscitation of trauma patients presenting in hemorrhagic shock [1, 2]. Studies have demonstrated that an increased transfusion ratio of plasma-to-red blood cells (RBCs) may prevent the development of trauma-induced coagulopathy and may also have the potential to improve the survival rate of severely injured patients [3–5]. However, other studies have failed to show any benefit of the high plasma-to-RBC transfusion ratio approach [6–8]. A recent randomized controlled trial also failed to show a clinically important difference in overall mortality between patients who received transfusion with a 1:1 blood product ratio of plasma and RBCs and those who received transfusion with a 1:2 ratio [9].

The scope of MT practices is mainly derived from studies in younger adults, and there is even less evidence showing the impact of early plasma transfusion practices on the elderly [10]. Elderly patients have generally reduced physiological reserve and an increased prevalence of comorbidities [11]. Higher transfusion ratios imply a larger total volume transfused, increasing the risk of complications, such as intravascular volume overload, transfusion-related lung injury, and infections [12–14]. A previous study using data from a Japanese administrative database, which included a large number of older adults, reported that a higher plasma-to-RBC ratio was associated with increased incidence of adverse events after MT in a ratio-dependent manner [15]. Although several investigators have documented increased mortality with increased age among trauma patients who received massive transfusions, the interaction between age and the effect of transfusion ratio has not been evaluated [16, 17].

This study aimed to assess the age-related heterogeneity in the association between plasma-to-RBC transfusion ratio and clinical outcomes in severely injured patients who required massive transfusion.

## Methods

### Study design and setting

This retrospective cohort study utilized the data from the American College of Surgeons Trauma Quality Improvement Program (TQIP) database. The TQIP database is a subset of the National Trauma Databank and contains trauma-related variables reported by trauma centers in the USA. The observation period was 4 years, from January 2013 to December 2016. At the end of 2016, more than 700 Level 1 and Level 2 trauma centers had participated in the TQIP. The study and its protocols were approved by the institutional review committee of Riverside University Health System—Comparative Effectiveness and Clinical Outcomes Research Center. The requirement for informed consent for each patient was waived based on the use of anonymized patient and hospital data.

### Patient selection

Severely injured patients who received massive transfusions were included in the analyses. To meet the TQIP inclusion criteria, patients were aged > 16 years, with an Abbreviated Injury Scale (AIS) score > 2 in at least one body region. Patients were excluded if their age, outcome, or length of hospital stay were unknown. We also excluded patients who arrived at the emergency department with no signs of life or died within the first 30 min of arrival [15, 18]. Patients who were transferred from another hospital or had isolated traumatic brain injury (AIS of the head > 2; AIS of other body parts, ≤ 2) were also excluded, since we aimed to assess the appropriate transfusion ratio in patients requiring massive transfusion due to hemorrhage.

### Data collection

The following variables were included in the study analyses: age, sex, comorbidities, mechanism of injury, Injury Severity Score (ISS), each body region’s AIS score, vital signs (systolic blood pressure, heart rate, and respiratory rate), Glasgow Coma Scale score upon arrival at the emergency department, hospital characteristics (trauma center level and teaching status), amount of blood transfusion within 4 h and 24 h (RBC, platelets, and plasma), hemostatic procedures, ICU length of stay and hospital length of stay, in-hospital complications, and in-hospital and 24-h mortality.

### Definitions and outcomes

A severely injured patient was defined as a patient with ISS ≥ 16. Massive transfusion was defined as transfusion of ≥ 5 units of RBC within 4 h of hospital admission or ≥ 10 units within 24 h of hospital admission, in accordance with previous studies [15, 17, 19–21]. Non-geriatric patients were defined as those aged between 16 and 64 years, and geriatric patients were defined as those aged ≥ 65 years. The study outcomes were in-hospital mortality rate, 24-h mortality rate, and incidence of pre-determined adverse events. The adverse events related to blood transfusion were defined as cardiac, respiratory, and renal failure, as well as thrombotic events and sepsis, which developed after hospital arrival.

### Statistical analysis

After selecting the study cohort, we performed robust linear regression analyses to statistically detect and remove outliers of plasma and RBC transfusion variables, considering the issue of registration errors in the values [22]. Missing values were then imputed using patient characteristics (age, sex), severity upon arrival to the emergency department (Revised Trauma Score [RTS], ISS, and AIS score of each body region), and comorbidities on arrival (cardiac, pulmonary, liver, or renal diseases, and diabetes) using the random forest method for each study cohort with the missForest package (version 1.4) of R software (version 3.5.2; R Foundation for Statistical Computing, Vienna, Austria) [23].

The associations between plasma-to-RBC transfusion ratios and outcomes were assessed as follows. First, we investigated the associations of the plasma-to-RBC ratio with in-hospital mortality, 24-h mortality, and incidence of adverse events in the entire study cohort using a non-linear generalized additive model (GAM), using the mgcv package (version 1.8-38) of R software, which was used to account for the possible non-linear relationship between the plasma-to-RBC ratio and outcomes based on the results of our previous study and preliminary analysis [15, 18]. The model was fitted using the residual maximum likelihood method, and adjusted for sex, ISS, RTS, injured body region, total prehospital time (time from injury to arrival at the hospital), trauma center level, and hospital type (university, community, and non-teaching). These variables were selected after considering a clinical perspective and previous reports [15, 18]. Second, we assessed the interaction of different age categories (i.e., non-geriatric patients or geriatric patients) with the association between the plasma-to-RBC ratio and in-hospital mortality of severely injured patients using a GAM. Third, the patients were classified into two age categories: non-geriatric and geriatric groups. Associations between the plasma-to-RBC ratio and outcomes (in-hospital mortality rate, 24-h mortality rate, and incidence of adverse events) were evaluated using a GAM adjusting for the aforementioned variables, which were used to visualize relationships between the plasma-to-RBC ratio and the outcomes in two different age groups. Fourth, the patients with different plasma-to-RBC ratios at 24 h were categorized into three groups: low (< 0.5), medium (0.5–1.0), and high (≥ 1.0). These three categories were chosen based on a clinical perspective and previous reports [18, 24]. Using a logistic regression model, the association between the plasma-to-RBC ratio categories and the outcomes was numerically compared between two consecutive categories (i.e., low vs. medium, medium vs. high, and low vs. high), adjusted for age, sex, comorbidities, injury mechanisms, total prehospital time, ISS, RTS and AIS in each body region (head, neck, thorax, abdomen, upper extremities, and pelvis/lower extremities), trauma center levels, and hospital type. We also performed Cox proportional hazard regression analyses after adjusting for age, sex, ISS, RTS, injured body region, and hospital types to further evaluate the risk of mortality at 28 days among the low, medium, and high subgroups in the non-geriatric and geriatric groups. Plasma-to-RBC ratios at 4 h and 24 h were treated as time-dependent covariates, and mortality after 28 days was treated as survival in this model [25]. The survival (version 3.2-13) and survminer (version 0.4.9) packages in R were used for the Cox proportional hazard regression analyses.

We performed sensitivity analyses using logistic regression models considering the possible effect of biases in observational studies on blood transfusion in trauma. First, we excluded all patients who died within the first 24 h to consider the impact of patients who died before plasma could be administered (survivor bias). Second, we included patients with similar plasma-to-RBC ratio categories between 4 and 24 h. For this analysis, we ensured that the transfusion strategy was consistent until 24 h by excluding patients whose transfusion ratio categories were different in the first 4 h vs. 24 h to reduce the risk of survivor and plasma delay bias [26]. Third, we carried out the same analysis for the cohort without severe traumatic brain injury (AIS of the head < 3), considering the different pathophysiology in patients with traumatic brain injuries [15].

Chi-square or Fisher’s exact test was used for categorical variables, and the Mann–Whitney *U* test was used for continuous variables. Values are presented as median (25–75% interquartile range) and frequency (percentage). Estimated data are presented as adjusted odds ratios (OR) with 95% confidence intervals (CI). All statistical analyses were performed using R software. The level of significance was defined as a *p* value < 0.05.

## Results

### Patients’ characteristics

**Fig. 1:**
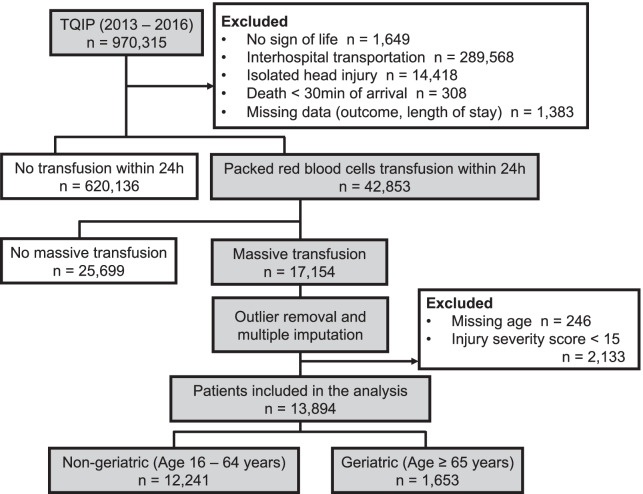
Diagramatic representation of patients who received massive transfusion in the Trauma Quality Improvement Program database

In total, 970,315 trauma patients were identified in the TQIP database between 2013 and 2016, of which 17,154 received ≥ 5 units of RBC within 4 h or ≥ 10 units of RBC within 24 h of hospital arrival. We excluded 3305 (19.2%) patients due to unknown age, outcome, or outliers of blood transfusion data. A total of 13,894 trauma patients received massive transfusions and met the inclusion criteria of the study, including 12,241 (88.1%) non-geriatric and 1653 (11.9%) geriatric patients (Fig. 1). Patients’ and hospital characteristics are summarized in Table 1. Geriatric patients were more likely to exhibit blunt mechanisms of injury and have more comorbidities. No significant differences in ISS and trauma center level between the study groups were observed. The in-hospital mortality rate was 30.5% for the entire study cohort, and it was significantly higher for geriatric patients than for non-geriatric patients (47.3% vs. 28.3%). The incidence of adverse events was similar among the two groups (22.8% vs. 21.9%) (Table 2).

**Table 1:** Description of patients and hospital characteristics

Description	Non-geriatric age group (*n* = 12,241)	Geriatric age group (*n* = 1653)	*p*-value
Patients’ characteristics			
Age, median (IQR)	33 (24–48)	72 (68–79)	< 0.001
Sex (male) *n* (%)	9647 (78.8)	1056 (65.5)	< 0.001
Mechanism of injury			< 0.001
Blunt injury, *n* (%)	9407 (76.8)	1422 (86.0)	
Penetrating injury, *n* (%)	2834 (23.2)	231 (14.0)	
Total prehospital time, median (IQR)	37 (24–63)	45 (31–69)	< 0.001
Comorbidities			
Heart failure, *n* (%)	45 (0.4)	59 (3.6)	< 0.001
Renal failure, *n* (%)	61 (0.5)	12 (0.7)	0.071
Cerebrovascular disease, *n* (%)	52 (0.4)	31 (1.9)	< 0.001
Diabetes, *n* (%)	546 (4.5)	264 (16.0)	< 0.001
Respiratory disease, *n* (%)	320 (2.6)	115 (7.0)	< 0.001
Liver cirrhosis, *n* (%)	181 (1.5)	35 (2.1)	0.062
Dementia, *n* (%)	7 (0.1)	41 (2.5)	< 0.001
SBP in the ED, median (IQR)	74 (60–90)	70 (57–83)	< 0.001
RTS, median (IQR)	6.1 (3.4–7.6)	6.8 (3.8–7.6)	< 0.001
AIS injured region			
Head ≥ 3, *n* (%)	3,989 (32.6)	634 (38.4)	< 0.001
Face ≥ 3, *n* (%)	371 (3.0)	39 (2.4)	0.151
Neck ≥ 3, *n* (%)	543 (4.4)	39 (2.4)	0.008
Thorax ≥ 3, *n* (%)	8481 (69.3)	1192 (76.2)	< 0.001
Abdomen ≥ 3, *n* (%)	7110 (58.1)	758 (47.2)	< 0.001
Spine ≥ 3, *n* (%)	1321 (10.8)	239 (13.5)	0.001
Upper extremity ≥ 3, *n* (%)	1067 (8.7)	86 (6.0)	< 0.001
Pelvis/lower extremity ≥ 3, *n* (%)	5383 (44.0)	792 (51.5)	< 0.001
Surface ≥ 3, *n* (%)	2 (0.001)	1 (0.001)	1.000
ISS, median (IQR)	29 (22–41)	29 (22–41)	0.677
Hospital characteristics			
Trauma center level			0.087
Level 1, *n* (%)	9108 (74.4)	1186 (71.7)	
Level 2, *n* (%)	3133 (25.6)	467 (28.3)	
Teaching status			0.004
University, *n* (%)	7602 (62.1)	957 (57.9)	
Community, *n* (%)	3752 (30.7)	565 (34.2)	
Non-teaching, *n* (%)	887 (7.2)	131 (7.9)	

*IQR* interquartile range; *SBP* systolic blood pressure; *ED* emergency department; *RTS* Revised Trauma Score; *AIS* Abbreviated Injury Scale; *ISS* Injury Severity Score

**Table 2:** Blood transfusion, hemorrhage control requirements, and outcomes

Variable	Non-geriatric age group (*n* = 12,241)	Geriatric age group (*n* = 1653)	*p*-value
Blood transfusion, units			
Within 4 h			
RBC, median (IQR)	9 (6–14)	8 (6–13)	< 0.001
Plasma, median (IQR)	6 (3–9)	4 (3–9)	< 0.001
Platelets, median (IQR)	1 (0–2)	1 (0–2)	0.024
Within 24 h			
RBC, median (IQR)	11 (7–16)	10 (7–16)	0.188
Plasma, median (IQR)	7 (4–12)	6 (4–11)	0.001
Platelets, median (IQR)	2 (1–3)	2 (1–3)	0.074
Hemorrhage control			
Angioembolization, *n* (%)	1713 (13.9)	337 (20.4)	< 0.001
Surgery, *n* (%)	9743 (79.6)	1128 (68.2)	< 0.001
Outcome			
In-hospital mortality, *n* (%)	3464 (28.3)	782 (47.3)	< 0.001
24-h mortality, *n* (%)	2231 (18.2)	430 (26.0)	< 0.001
Hospital LOS, median (IQR)	14 (3–27)	9 (1–22)	< 0.001
ICU admission, *n* (%)	10,641 (86.9)	1444 (87.3)	0.668
ICU LOS, median (IQR)	8 (2–12)	7 (2–16)	0.157
Ventilator days, median (IQR)	4 (2–12)	4 (1–12)	0.443
Any adverse events, *n* (%)	2685 (21.9)	377 (22.8)	0.440
Cardiac failure, *n* (%)	43 (0.4)	44 (2.7)	< 0.001
Respiratory failure, *n* (%)	698 (5.7)	82 (5.0)	0.241
Renal failure, *n* (%)	856 (7.0)	136 (8.2)	0.075
Thrombosis, *n* (%)	1337 (11.0)	134 (12.2)	0.102
Sepsis, *n* (%)	370 (3.0)	37 (3.0)	1.000
Others, *n* (%)	1052 (8.6)	149 (9.0)	0.412

*RBC* red blood cell; *IQR* interquartile range; *LOS* length of stay; *ICU* intensive care unit

### Association between the plasma-to-RBC ratio and the outcomes

**Fig. 2:**
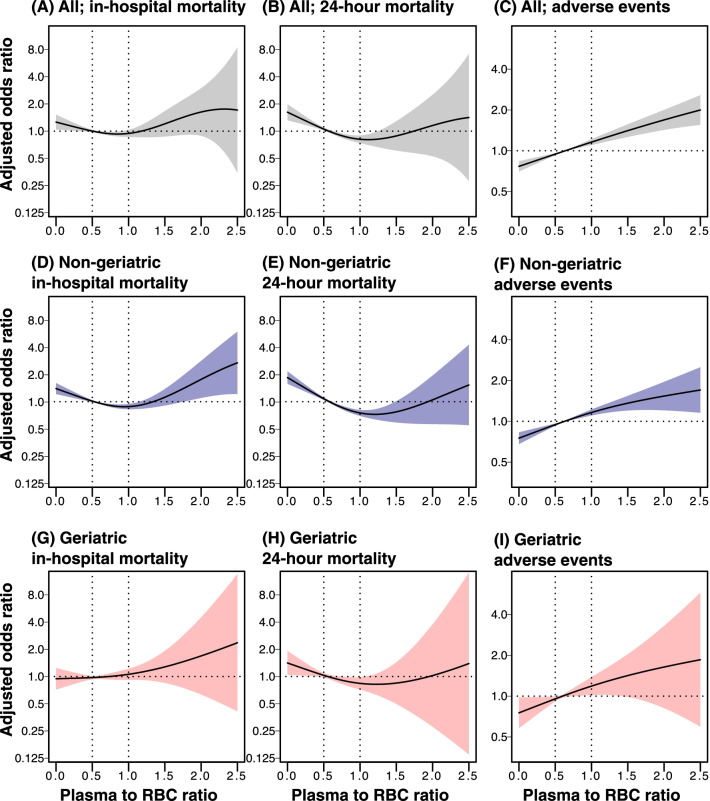
Generalized additive model evaluating the association between plasma-to-red blood cell ratios and outcomes. Association of plasma-to-red blood cell ratios and study outcomes in the entire cohort (**A–C**) and in the non-geriatric group (**D–F**) and geriatric group (**G–I**). Mortality and adverse events were analyzed using the non-linear logistic generalized additive model adjusted for sex, injury mechanisms, Revised Trauma Score, Injury Severity Score, and Abbreviated Injury Scale Score in each body region (head, neck, thorax, abdomen, upper extremities, and pelvis/lower extremities), total prehospital time, trauma center level, and hospital type (university, community, and non-teaching). Adverse events included cardiac, respiratory, and renal failure, as well as thrombotic events and sepsis. The shaded region represents the 95% confidence intervals for the estimated points

Using a non-linear GAM to analyze the entire cohort, the plot for assessing the in-hospital and 24-h mortality rates after adjusting for multiple covariables demonstrated a downward convex unimodal curve (Fig. 2A, B). A trend toward a higher incidence of adverse events was observed for higher plasma-to-RBC ratios (Fig. 2C). We then confirmed that the interaction between the age category and plasma-to-RBC ratio at 24 h was statistically significant (*p* for interaction < 0.001), suggesting the existence of an interaction between age categorization and the plasma-to-RBC ratio with mortality.

After patients were subdivided into two age categories, the GAM plots of the plasma-to-RBC ratio for both in-hospital and 24-h mortality rates demonstrated a downward convex unimodal curve in the non-geriatric group (Fig. 2D, E). However, the plot showed no specific threshold in the risk of in-hospital mortality among the geriatric group (Fig. 2G). A trend toward increased incidence of adverse events was observed for higher plasma-to-RBC ratios in both non-geriatric and geriatric groups (Fig. 2F, I).

Considering generalizability and the results of the GAM plots, we divided the plasma-to-RBC ratios into the following three categories: low (< 0.5), medium (0.5–1.0), and high (≥ 1.0). In the categorized multivariate regression analysis, the low ratio group was associated with higher in-hospital and 24-h mortality rates than the medium ratio group among the non-geriatric patients (Table 3 and Additional file 1: Table S1). However, there was no association between the transfusion ratio and mortality rate in the geriatric group (Table 3). As per the GAM plots, an increase in the plasma-to-RBC ratio was significantly associated with increased incidence of adverse events in both the non-geriatric and geriatric groups and between the low and medium ratio groups (Table 3).

**Table 3:** Logistic regression analysis evaluating outcomes according to plasma-to-RBC ratio categories

Plasma-to-RBC ratio category	Non-geriatric group			Geriatric group		
	*n*	Age: 16–64 years	*p*	*n*	Age: ≥ 65 years	*p*
		OR (95% CI)			OR (95% CI)	
In-hospital mortality						
Low	4239	1.38 (1.22–1.56)	< 0.001	642	0.84 (0.62–1.12)	0.233
Medium	6938	1.00 [Reference]		897	1.00 [Reference]	
High	1064	1.21 (0.98–1.49)	0.149	114	1.34 (0.80–2.27)	0.276
24-h mortality						
Low	4239	1.75 (1.50–2.00)	< 0.001	642	1.11 (0.79–1.55)	0.522
Medium	6938	1.00 [Reference]		897	1.00 [Reference]	
High	1064	0.92 (0.71–1.18)	0.57	114	0.85 (0.42–1.63)	0.657
Adverse events						
Low	4239	0.70 (0.62–0.80)	< 0.001	642	0.6 (0.43–0.84)	0.002
Medium	6938	1.00 [Reference]		897	1.00 [Reference]	
High	1064	1.16 (0.96–1.39)	0.111	114	0.79 (0.43–1.40)	0.438

Patients were stratified according to plasma-to-RBC ratio as follows: low < 0.5, medium 0.5–1.0, and high ≥ 1.0. The model was adjusted for age, sex, comorbidities, injury mechanisms, total prehospital time, Revised Trauma Score, Injury Severity Score, and Abbreviated Injury Scale score, in each body region (head, neck, thorax, abdomen, upper extremities, and pelvis/lower extremities), trauma center levels, and hospital types (university, community, and non-teaching). Adverse events; cardiac failure, respiratory failure, acute renal failure, thrombotic events, and sepsis

*RBC* red blood cell

**Fig. 3:**
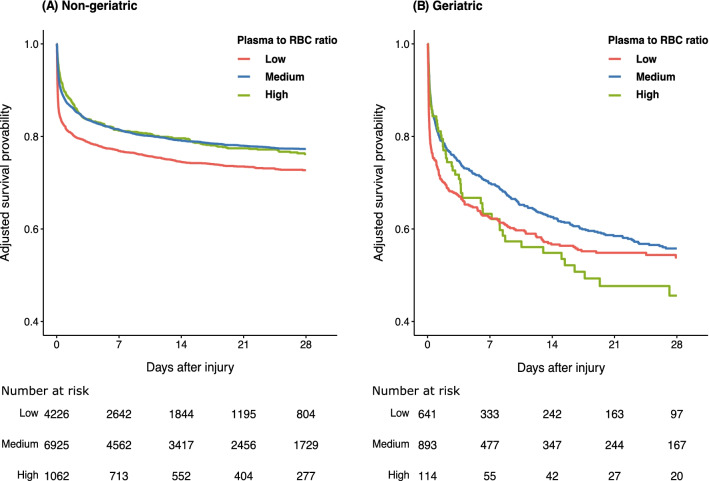
Cumulative survival curves using Cox proportional hazard regression model with a time-dependent covariate. The cumulative survival curves for the low (< 0.5), medium (0.5–1.0), and high (≥ 1.0) plasma-to-red blood cell transfusion ratio groups, which were estimated using the Cox proportional hazard regression model for non-geriatric group (**A**) and geriatric group (**B**) patients. The hazard ratio for 28-day mortality was controlled for age, sex, injury severity score, Revised Trauma Score, injured region, and hospital type. The plasma-to-RBC ratios at 4 h and 24 h were treated as time-dependent covariates

A multivariate Cox proportional hazard regression analysis revealed that the low plasma-to-RBC transfusion ratio was associated with a higher risk of 28-day mortality (hazard ratio 1.13, 95% CI 1.05–1.22, *p* < 0.001) compared to the medium ratio after controlling for multiple confounders in the non-geriatric patients. A similar association was lacking for the medium ratio group of geriatric patients (Fig. 3 and Table 4).

**Table 4:** Cox proportional hazard regression analysis of transfusion ratio categories with 28-day mortality

Plasma-to-RBC ratio category	Non-geriatric group			Geriatric group		
	n	Age: 16–64 years	*p*	n	Age: ≥ 65 years	*p*
		HR (95% CI)			HR (95% CI)	
Low	4,226	1.13 (1.05–1.22)	< 0.001	641	0.94 (0.92–1.21)	0.396
Medium	6,925	1.00 [Reference]		893	1.00 [Reference]	
High	1,062	1.07 (0.93–1.20)	0.383	114	0.92 (0.72–1.18)	0.53

Patients were stratified according to the plasma-to-RBC ratio as follows: low < 0.5, medium 0.5–1.0, and high ≥ 1.0. Cox proportional hazard regression analysis controlling for age, sex, Injury Severity Scale, Revised Trauma Score, injured body region, and hospital types to evaluate the risk of mortality at 28 days between low, medium, and high groups in non-geriatric and geriatric groups. Plasma-to-RBC ratios at 4 and 24 h were treated as time-dependent covariates, and mortality for > 28 days was treated as survival in this model

*RBC* red blood cell

The sensitivity analyses showed that the association of a low plasma-to-RBC ratio with in-hospital mortality rate remained significantly higher in the subsets of 10,359 patients, whose plasma-to-RBC ratio category was the same at 4 h and 24 h, as well as for the 8252 patients without severe traumatic brain injury in the non-geriatric group (Additional file 2: Table 2). After excluding patients who died within the first 24 h, the association between the plasma-to-RBC ratios and in-hospital mortality rate was diminished in the non-geriatric group (OR vs. medium group, 0.98; 95% CI 0.56–1.51) (Additional file 2: Table S2). In accordance with the entire cohort, significant correlations between transfusion ratios and in-hospital mortality were absent in all sub-populations of the geriatric group (Additional file 2: Table S2).

## Discussion

In this study, we evaluated the association between plasma-to-RBC ratios and outcomes in severely injured geriatric and non-geriatric patients using a nationwide trauma database. We observed that patients who received massive transfusion with a low plasma-to-RBC ratio had significantly greater odds of mortality than patients who received plasma transfusion with a ratio in the range of 0.5–1.0 among non-geriatric severely injured patients. However, an apparent trend in mortality according to the plasma-to-RBC ratio was absent in the geriatric group. Furthermore, an increased incidence of adverse events was observed in the geriatric and non-geriatric trauma patients who received massive transfusions with higher plasma-to-RBC ratios.

Transfusions with balanced plasma-to-RBC ratios as close to 1:1 have been reported to be associated with a reduction in the risk of death from hemorrhage [4, 27, 28]; however, several early observational studies on blood product ratios might have been affected by survival or selection biases. A meta-analysis conducted by a committee of the Eastern Association for the Surgery of Trauma could not provide high-grade evidence supporting the survival benefit of a higher plasma-to-RBC transfusion strategy [2]. The PROPPR trial, the only large randomized controlled trial comparing outcomes between patients who received transfusion in either 1:1:1 or 1:1:2 ratio of plasma, platelets, and RBC, was unable to demonstrate a statistically significant difference between the 24-h and 30-day mortality rates [9]. Therefore, the conclusive optimal plasma-to-RBC transfusion ratio remains undetermined.

Based on the results of this study, we postulated that the results of previous studies might have been affected by the heterogeneity of the analyzed participants in terms of injury mechanisms and surgical interventions, as well as patient characteristics [2, 29, 30]. Hence, we focused on the differences in the association of transfusion ratios and mortality rate according to age, since advanced age is an independent risk factor for mortality after massive transfusion [17, 31, 32]. Although Cannon et al. reported that the impact of plasma-to-RBC ratio on the mortality of severely injured patients was affected by age, their study targeted a pediatric population [29]. To the best of our knowledge, this is the first study to examine the association of age (whether geriatric or not) with transfusion product ratios and mortality.

Plasma transfusions can cause several adverse events, including, but not limited to, transfusion-associated circulatory overload, transfusion-related acute lung injury, allergic reactions, and transfusion-transmitted viral infections, suggesting that excessive administration of plasma might potentially increase the risk of adverse outcomes [12–14]. A previous large-scale retrospective study analyzing a Japanese administrative database failed to show significant differences in in-hospital mortality and transfusion strategies among low (< 0.75), medium (0.75–1.25), and high (≥ 1.25) plasma-to-RBC ratios, and the high plasma-to-RBC ratio strategy was associated with an increase in adverse events [15]. However, there was no association between the plasma-to-RBC ratio and transfusion-related complications in the PROPPR trial [9]. These observed differences may be partially explained by the participants’ age in the cohort: median age was 61 years and 34 years in the Japanese study and PROPPR trial, respectively. Although a considerable number of studies have also reported a higher incidence of adverse events after massive transfusion in older patients, the association between the plasma-to-RBC ratio and adverse events has not been investigated in that subgroup of older patients [10, 31, 33, 34]. In this study, an association between the plasma-to-RBC transfusion ratio and mortality was not observed in the geriatric trauma population. Alternative transfusion strategies for injured geriatric patients, such as developing prediction scores for massive transfusion requirements or point-of-care-based coagulation tests, may be required to improve their outcomes and minimize the risk of transfusion-related complications [35, 36].

Geriatric trauma has become increasingly common as the population ages, particularly in several developed countries [37]. A body of evidence has documented significantly worse outcomes in older patients compared to their younger counterparts [32, 37, 38]. However, geriatric-specific data on blood transfusion ratios during trauma-related resuscitation are scarce. Although recent studies have reported that early aggressive transfusion of plasma is associated with better outcomes than delayed transfusion, others have also raised concerns regarding the safety of increased plasma transfusion, citing the increased risk of complications [39]. In our study, there was no significant association among the different plasma transfusion ratios with mortality of geriatric patients, whereas a low plasma-to-RBC transfusion ratio was associated with a higher risk of 28-day mortality in the non-geriatric patients. One possible explanation is that the effect size of plasma-to-RBC ratio is lower among elderly patients than younger patients due to the reduced physiological reserve and high prevalence of pre-existing comorbidities [11, 40]. Although some other researchers have advocated a restrictive blood transfusion protocol for geriatric trauma patients, the benefit and adverse events of this strategy must be further evaluated in future studies [41, 42]. Another possible reason is the “Do Not Attempt Resuscitation” (DNAR) order. In general, DNAR orders are more often in geriatric patients [43, 44]. This trend might also have reduced the effect size of plasma-to-RBC ratio in this study.

One of the strengths of this study was that we confirmed the association between the transfusion ratio and mortality using a time-dependent Cox regression model. Survival bias could be reduced by treating the plasma-to-RBC ratio as a time-dependent covariate. We also confirmed consistent results in the sub-analysis on a cohort with a similar transfusion ratio at 4-h and 24-h time points and on a cohort without severe head injury.

The limitations of this study should be acknowledged. First, since this was a retrospective analysis, the presence of survival bias cannot be excluded. Fresh frozen plasma takes longer to administer than RBC, and some patients with devastating conditions might have died before receiving adequate plasma volume. We excluded patients who died within 30 min of arrival at the emergency department, thus aiming to reduce some of the potential survival bias. Second, the TQIP database does not contain detailed information about medications administered to trauma patients. Geriatric patients are more likely to take anticoagulants or antiplatelet agents, which can be a confounding factor for plasma transfusion and outcomes. Third, we compared the outcomes between heterogeneous groups of geriatric and non-geriatric trauma patients. The number of non-geriatric patients age ≥ 65 years was relatively small, as with any trauma database, and this could lead to a type II error. Fourth, a controversy exists regarding the definition of massive transfusions. Massive transfusion is commonly defined as administration of ≥ 10 units of RBC within the first 24 h, as used in this study [19]. However, several researchers have raised concerns regarding this traditional definition [45–47]. Alternatively, others have defined massive transfusion as a transfusion of ≥ 3–4 units of RBC during the initial phase of resuscitation, which is mainly within the first hour [45, 47, 48]. The TQIP database includes the amount of blood products administered within 4 h of admission, but not in the first hour. Therefore, we used a substitute criterion (≥ 5 units of RBC in 4 h), which has been used in previous studies [20, 21]. DNR orders are important variables that affect the outcomes and transfusion strategies in trauma patients [43, 44]. A lack of this information can also be considered a limitation of the study. Finally, since we used a national data set from a single country, external validation may be needed.

## Conclusions

In conclusion, age interacted with the association between the plasma-to-RBC ratio and in-hospital mortality in severely injured patients who underwent massive transfusions. Massive transfusion with a low plasma-to-RBC ratio (< 0.5) was not beneficial, especially in non-geriatric adult trauma patients. The association between the plasma-to-RBC ratio and mortality among geriatric patients remains inconclusive.

## Supplementary Information

**Additional file 1: Table S1.** Results of logistic regression analysis for outcomes according to plasma-to-red blood cell ratio categories.

**Additional file 2: Table S2.** Logistic regression model for in-hospital mortality among sub-populations, excluding the following patients from the entire cohort.

## Data Availability

All data used in this study are available from the American College of Surgeons Trauma Quality Improvement Program.

## Abbreviations

AIS: Abbreviated Injury Scale
CI: Confidence intervals
GAM: Generalized additive model
ISS: Injury Severity Score
OR: Odds ratios
PROPPR: Pragmatic, Randomized Optimal Platelet and Plasma Ratios
RBC: Red blood cell
TQIP: Trauma Quality Improvement Program

## References

[1] Kalkwarf KJ, Cotton BA (2017). Resuscitation for hypovolemic shock. Surg Clin North Am.

[2] Cannon JW, Khan MA, Raja AS, Cohen MJ, Como JJ, Cotton BA (2017). Damage control resuscitation in patients with severe traumatic hemorrhage: a practice management guideline from the Eastern Association for the Surgery of Trauma. J Trauma Acute Care Surg.

[3] Gonzalez E, Moore EE, Moore HB, Chapman MP, Chin TL, Ghasabyan A (2016). Goal-directed hemostatic resuscitation of trauma-induced coagulopathy: a pragmatic randomized clinical trial comparing a viscoelastic assay to conventional coagulation assays. Ann Surg.

[4] Roberts DJ, Ball CG, Feliciano DV, Moore EE, Ivatury RR, Lucas CE (2017). History of the innovation of damage control for management of trauma patients: 1902–2016. Ann Surg.

[5] Stewart RM, Park PK, Hunt JP, McIntyre RC, Jr., McCarthy J, Zarzabal LA, et al. Less is more: improved outcomes in surgical patients with conservative fluid administration and central venous catheter monitoring. J Am Coll Surg. 2009;208:725–35; discussion 35–7. 10.1016/j.jamcollsurg.2009.01.026. 10.1016/j.jamcollsurg.2009.01.026 19476825

[6] Halmin M, Boström F, Brattström O, Lundahl J, Wikman A, Östlund A (2013). Effect of plasma-to-RBC ratios in trauma patients: a cohort study with time-dependent data. Crit Care Med.

[7] Snyder CW, Weinberg JA, McGwin G, Melton SM, George RL, Reiff DA (2009). The relationship of blood product ratio to mortality: survival benefit or survival bias?. J Trauma.

[8] Sambasivan CN, Kunio NR, Nair PV, Zink KA, Michalek JE, Holcomb JB (2011). High ratios of plasma and platelets to packed red blood cells do not affect mortality in nonmassively transfused patients. J Trauma.

[9] Holcomb JB, Tilley BC, Baraniuk S, Fox EE, Wade CE, Podbielski JM (2015). Transfusion of plasma, platelets, and red blood cells in a 1:1:1 vs a 1:1:2 ratio and mortality in patients with severe trauma: the PROPPR randomized clinical trial. JAMA.

[10] Mador B, Nascimento B, Hollands S, Rizoli S (2017). Blood transfusion and coagulopathy in geriatric trauma patients. Scand J Trauma Resusc Emerg Med.

[11] Llompart-Pou JA, Pérez-Bárcena J, Chico-Fernández M, Sánchez-Casado M, Raurich JM (2017). Severe trauma in the geriatric population. World J Crit Care Med.

[12] Vlaar AP, Juffermans NP (2013). Transfusion-related acute lung injury: a clinical review. Lancet.

[13] Alcorn K, Ramsey G, Souers R, Lehman CM (2017). Appropriateness of plasma transfusion: a college of American pathologists Q-probes study of guidelines, waste, and serious adverse events. Arch Pathol Lab Med.

[14] Clifford L, Jia Q, Subramanian A, Yadav H, Schroeder DR, Kor DJ (2017). Risk factors and clinical outcomes associated with perioperative transfusion-associated circulatory overload. Anesthesiology.

[15] Endo A, Shiraishi A, Fushimi K, Murata K, Otomo Y (2018). Outcomes of patients receiving a massive transfusion for major trauma. Br J Surg.

[16] Hashmi A, Ibrahim-Zada I, Rhee P, Aziz H, Fain MJ, Friese RS (2014). Predictors of mortality in geriatric trauma patients: a systematic review and meta-analysis. J Trauma Acute Care Surg.

[17] Mitra B, Olaussen A, Cameron PA, O'Donohoe T, Fitzgerald M (2014). Massive blood transfusions post trauma in the elderly compared to younger patients. Injury.

[18] Butler EK, Mills BM, Arbabi S, Bulger EM, Vavilala MS, Groner JI (2019). Association of blood component ratios with 24-hour mortality in injured children receiving massive transfusion. Crit Care Med.

[19] Trickey AW, Fox EE, del Junco DJ, Ning J, Holcomb JB, Brasel KJ (2013). The impact of missing trauma data on predicting massive transfusion. J Trauma Acute Care Surg.

[20] Mitra B, Cameron PA, Gruen RL, Mori A, Fitzgerald M, Street A (2011). The definition of massive transfusion in trauma: a critical variable in examining evidence for resuscitation. Eur J Emerg Med.

[21] Mitra B, Mori A, Cameron PA, Fitzgerald M, Street A, Bailey M (2007). Massive blood transfusion and trauma resuscitation. Injury.

[22] Shiraishi A, Otomo Y, Yoshikawa S, Morishita K, Roberts I, Matsui H (2019). Derivation and validation of an easy-to-compute trauma score that improves prognostication of mortality or the Trauma Rating Index in Age, Glasgow Coma Scale, Respiratory rate and Systolic blood pressure (TRIAGES) score. Crit Care.

[23] Stekhoven DJ, Bühlmann P (2012). MissForest—non-parametric missing value imputation for mixed-type data. Bioinformatics.

[24] Holcomb JB, del Junco DJ, Fox EE, Wade CE, Cohen MJ, Schreiber MA (2013). The prospective, observational, multicenter, major trauma transfusion (PROMMTT) study: comparative effectiveness of a time-varying treatment with competing risks. JAMA Surg.

[25] Brown JB, Cohen MJ, Minei JP, Maier RV, West MA, Billiar TR, et al. Debunking the survival bias myth: characterization of mortality during the initial 24 hours for patients requiring massive transfusion. J Trauma Acute Care Surg. 2012;73:358–64; discussion 64. 10.1097/TA.0b013e31825889ba. 10.1097/TA.0b013e31825889ba 22846940

[26] Nederpelt CJ, El Hechi MW, Kongkaewpaisan N, Kokoroskos N, Mendoza AE, Saillant NN (2020). Fresh frozen plasma-to-packed red blood cell ratio and mortality in traumatic hemorrhage: nationwide analysis of 4,427 patients. J Am Coll Surg.

[27] Palm K, Apodaca A, Spencer D, Costanzo G, Bailey J, Blackbourne LH (2012). Evaluation of military trauma system practices related to damage-control resuscitation. J Trauma Acute Care Surg.

[28] Borgman MA, Spinella PC, Perkins JG, Grathwohl KW, Repine T, Beekley AC (2007). The ratio of blood products transfused affects mortality in patients receiving massive transfusions at a combat support hospital. J Trauma.

[29] Cannon JW, Johnson MA, Caskey RC, Borgman MA, Neff LP (2017). High ratio plasma resuscitation does not improve survival in pediatric trauma patients. J Trauma Acute Care Surg.

[30] Boutin A, Chasse M, Shemilt M, Lauzier F, Moore L, Zarychanski R (2016). Red blood cell transfusion in patients with traumatic brain injury: a systematic review and meta-analysis. Transfus Med Rev.

[31] DeLeon AN, Uecker JM, Stafford SV, Ali S, Clark A, Brown CV (2016). Restrictive transfusion in geriatric trauma patients. Am Surg.

[32] Hwabejire JO, Nembhard CE, Oyetunji TA, Seyoum T, Abiodun MP, Siram SM (2017). Age-related mortality in blunt traumatic hemorrhagic shock: the killers and the life savers. J Surg Res.

[33] Murphy MF, Estcourt L, Goodnough LT (2017). Blood transfusion strategies in elderly patients. Lancet Haematol.

[34] Putot A, Zeller M, Perrin S, Beer JC, Ravisy J, Guenancia C (2018). Blood transfusion in elderly patients with acute myocardial infarction: data from the RICO Survey. Am J Med.

[35] Schöchl H, Cotton B, Inaba K, Nienaber U, Fischer H, Voelckel W (2011). FIBTEM provides early prediction of massive transfusion in trauma. Crit Care.

[36] Theusinger OM, Stein P, Levy JH (2015). Point of care and factor concentrate-based coagulation algorithms. Transfus Med Hemother.

[37] Kojima M, Endo A, Shiraishi A, Otomo Y (2019). Age-related characteristics and outcomes for patients with severe trauma: analysis of Japan's Nationwide Trauma Registry. Ann Emerg Med.

[38] Brown CVR, Rix K, Klein AL, Ford B, Teixeira PGR, Aydelotte J (2016). A comprehensive investigation of comorbidities, mechanisms, injury patterns, and outcomes in geriatric blunt trauma patients. Am Surg.

[39] Hagiwara A, Kushimoto S, Kato H, Sasaki J, Ogura H, Matsuoka T (2016). Can early aggressive administration of fresh frozen plasma improve outcomes in patients with severe blunt trauma?—a report by the Japanese Association for the Surgery of Trauma. Shock.

[40] Murry JS, Zaw AA, Hoang DM, Mehrzadi D, Tran D, Nuno M, et al. Activation of massive transfusion for elderly trauma patients. Am Surg. 2015;81:945–9. 10.1177/000313481508101007. 26463286

[41] Keuter K, Ablah E, Vasquez D, Wetta-Hall R, Hawley SR (2008). Blood transfusions in elderly trauma patients: is there a role for restrictive use?. J Am Geriatr Soc.

[42] Loftus TJ, Brakenridge SC, Murphy TW, Nguyen LL, Moore FA, Efron PA (2018). Anemia and blood transfusion in elderly trauma patients. J Surg Res.

[43] Adams SD, Cotton BA, Wade CE, Kozar RA, Dipasupil E, Podbielski JM (2013). Do not resuscitate status, not age, affects outcomes after injury: an evaluation of 15,227 consecutive trauma patients. J Trauma Acute Care Surg.

[44] Wade CE, del Junco DJ, Fox EE, Cotton BA, Cohen MJ, Muskat P (2013). Do-not-resuscitate orders in trauma patients may bias mortality-based effect estimates: an evaluation using the PROMMTT study. J Trauma Acute Care Surg.

[45] Savage SA, Sumislawski JJ, Croce MA, Zarzaur BL (2014). Using critical administration thresholds to predict abbreviated laparotomy. J Trauma Acute Care Surg.

[46] Nunez TC, Young PP, Holcomb JB, Cotton BA (2010). Creation, implementation, and maturation of a massive transfusion protocol for the exsanguinating trauma patient. J Trauma.

[47] Savage SA, Sumislawski JJ, Zarzaur BL, Dutton WP, Croce MA, Fabian TC. The new metric to define large-volume hemorrhage: results of a prospective study of the critical administration threshold. J Trauma Acute Care Surg. 2015;78:224–9; discussion 9–30. 10.1097/TA.0000000000000502. 10.1097/TA.0000000000000502 25757105

[48] Moren AM, Hamptom D, Diggs B, Kiraly L, Fox EE, Holcomb JB (2015). Recursive partitioning identifies greater than 4 U of packed red blood cells per hour as an improved massive transfusion definition. J Trauma Acute Care Surg.

