# Supplementary material for: Association between the plasma-to-red blood cell ratio and survival in geriatric and non-geriatric trauma patients undergoing massive transfusion: a retrospective cohort study
Source: J Intensive Care. 2022 Jan 11;10:2. doi: 10.1186/s40560-022-00595-7 (PMC8753889; doi:10.1186/s40560-022-00595-7)
Supplement: Supplementary file 1 — Additional file 1: Table S1. Results of logistic regression analysis for outcomes according to plasma-to-red blood cell ratio categories. [file 40560_2022_595_MOESM1_ESM.docx]

**Additional file 1.** **Logistic regression analysis for outcomes according to plasma to RBC ratio categories**

|  |  | | **Non-geriatrics** Age: 16-64 years | | | | | | | |  | | |  | | | **Geriatrics** Age ≥ 65 years | | | | | | | | | |  |
| --- | --- | --- | --- | --- | --- | --- | --- | --- | --- | --- | --- | --- | --- | --- | --- | --- | --- | --- | --- | --- | --- | --- | --- | --- | --- | --- | --- |
|  |  | |  |  |  |  |  |  |  |  |  | | |  | | |  |  |  |  |  |  |  |  |  |  |  |
| Plasma to RBC ratio | n | | | | OR | 95% CI | | | p | | | |  | | | n | | | OR | | 95% CI | | p | | | | |
| **In-hospital mortality** | | | | |  |  | | |  | | | |  | | |  | | |  | |  | |  | | | | |
| **Low** | 4239 | | | | 1.00 [Reference] | | | | | | | |  | | 642 | | | | 1.00 [Reference] | | | | | | |  |  |
| **Medium** | 6938 | | | | 0.74 | (0.65–0.83) | | < 0.001 | | | | |  | | | 897 | | | 1.19 | | (0.89–1.59) | | 0.249 | | | | |
| **High** | 1064 | | | | 0.89 | (0.72–1.10) | | 0.292 | | | | |  | | 114 | | | | 2.15 | | (1.20–3.87) | | 0.009 | | |  |  |
| **24-hour mortality** | |  | |  | | |  |  | |  | | |  | | | | |  | | | |  | | |  |  |  |
| **Low** | 4239 | | | | 1.00 [Reference] | | | | | | | |  | | 642 | | | | 1.00 [Reference] | | | | | | |  |  |
| **Medium** | 6938 | | | | 0.59 | (0.52–0.68) | | < 0.001 | | | | |  | | | 897 | | | 0.88 | | (0.64–1.23) | | 0.458 | | | | |
| **High** | 1064 | | | | 0.50 | (0.38–0.65) | | < 0.001 | | | | |  | | 114 | | | | 0.93 | | (0.44–1.90) | | 0.859 | | |  |  |
| **Adverse events** |  | | | |  |  | |  | | | |  | | | |  | | | |  |  | | |  | |  |  |
| **Low** | 4239 | | | | 1.00 [Reference] | | | | | | | |  | | 642 | | | | 1.00 [Reference] | | | | | | |  |  |
| **Medium** | 6938 | | | | 1.39 | (1.25-1.59) | | <0.001 | | | | |  | | | 897 | | | 1.67 | | (1.19-2.33) | | 0.025 | | | |  |
| **High** | 1064 | | | | 1.71 | (1.39-2.08) | | <0.001 | | | | |  | | 114 | | | | 1.19 | | (0.62-2.22) | | 0.577 | | |  |  |

Patients were stratified according to plasma to red blood cells ratio as follows: low, 0⋅5 or less; medium, over 0.5 to 1.0; high, over 1.0. The model was adjusted for age, sex, comorbidities, injury mechanisms, Revised Trauma Score, Injury Severity Score, Abbreviated Injury Scale in each body region (head, neck, thorax, abdomen, upper extremities and pelvis/lower extremities), trauma center levels, and hospital types (university, community, and non-teaching). Adverse events: cardiac failure, respiratory failure, acute renal failure, thrombotic events, and sepsis.

RBC, red blood cells; OR, adjusted odds ratio; CI, confidence interval.
