# Supplementary material for: Association between the plasma-to-red blood cell ratio and survival in geriatric and non-geriatric trauma patients undergoing massive transfusion: a retrospective cohort study
Source: J Intensive Care. 2022 Jan 11;10:2. doi: 10.1186/s40560-022-00595-7 (PMC8753889; doi:10.1186/s40560-022-00595-7)
Supplement: Supplementary file 2 — Additional file 2: Table S2. Logistic regression model for in-hospital mortality among sub-populations, excluding the following patients from the entire cohort. [file 40560_2022_595_MOESM2_ESM.docx]

**Additional file 2.** **Logistic regression model for in-hospital mortality among subpopulations excluding following patients from the entire cohort.**

1. Excluding patients who died within 24 hours

|  |  | **Non-geriatrics** Age: 16-64 years | | | | |  | | |  | | | **Geriatrics** Age ≥ 65 years | | | | | | |  |  |
| --- | --- | --- | --- | --- | --- | --- | --- | --- | --- | --- | --- | --- | --- | --- | --- | --- | --- | --- | --- | --- | --- |
|  |  |  |  |  |  |  |  | | |  | | |  |  |  |  |  |  |  |  |  |
| Plasma to RBC ratio | n | | OR | 95% CI | | p | | |  | | | n | | | OR | | 95% CI | p | | |  |
| **Low** | 3179 | | 0.93 | (0.76–1.12) | 0.411 | | |  | | | 434 | | | 0.68 | | (0.46–0.99) | | | 0.045 | | |
| **Medium** | 5524 | | 1.00 [Reference] | | | | | |  | | | 630 | | | 1.00 [Reference] | | | | | |  |
| **High** | 871 | | 1.43 | (1.09–1.86) | 0.007 | | |  | | | 86 | | | 1.07 | | (0.93–1.20) | | | 0.383 | | |

1. Excluding patients whose plasma-to-RBC ratio categories were different at 4 and 24 hours

|  |  | **Non-geriatrics** Age: 16-64 years | | | | |  | | |  | | | **Geriatrics** Age ≥ 65 years | | | | | | |  |  |
| --- | --- | --- | --- | --- | --- | --- | --- | --- | --- | --- | --- | --- | --- | --- | --- | --- | --- | --- | --- | --- | --- |
|  |  |  |  |  |  |  |  | | |  | | |  |  |  |  |  |  |  |  |  |
| Plasma to RBC ratio | n | | OR | 95% CI | | p | | |  | | | n | | | OR | | 95% CI | p | | |  |
| **Low** | 3888 | | 1.40 | (1.23-1.60) | <0.001 | | |  | | | 576 | | | 0.79 | | (0.57-1.09) | | | 0.152 | | |
| **Medium** | 5806 | | 1.00 [Reference] | | | | | |  | | | 681 | | | 1.00 [Reference] | | | | | |  |
| **High** | 665 | | 1.07 | (0.93-1.20) | 0.383 | | |  | | | 69 | | | 1.67 | | (0.82-3.42) | | | 0.161 | | |

1. Excluding patients with severe traumatic brain injury

|  |  | **Non-geriatrics** Age: 16-64 years | | | | |  | | |  | | | **Geriatrics** Age ≥ 65 years | | | | | | |  |  |
| --- | --- | --- | --- | --- | --- | --- | --- | --- | --- | --- | --- | --- | --- | --- | --- | --- | --- | --- | --- | --- | --- |
|  |  |  |  |  |  |  |  | | |  | | |  |  |  |  |  |  |  |  |  |
| Plasma to RBC ratio | n | | OR | 95% CI | | p | | |  | | | n | | | OR | | 95% CI | p | | |  |
| **Low** | 2886 | | 1.65 | (1.41–1.96) | <0.001 | | |  | | | 404 | | | 0.94 | | (0.64–1.36) | | | 0.189 | | |
| **Medium** | 4678 | | 1.00 [Reference] | | | | | |  | | | 548 | | | 1.00 [Reference] | | | | | |  |
| **High** | 688 | | 1.66 | (0.82–3.42) | 0.161 | | |  | | | 67 | | | 0.92 | | (0.44–1.88) | | | 0.835 | | |

Patients were stratified according to plasma to red blood cells ratio: low, 0⋅5 or less; medium, over 0.5 to 1.0; high, over 1.0. The model was adjusted for age, sex, comorbidities, injury mechanisms, Revised Trauma Score, Injury Severity Score, Abbreviated Injury Scale in each body region (head, neck, thorax, abdomen, upper extremities, and pelvis/lower extremities), trauma center levels, and hospital types (university, community, and non-teaching).

RBC, red blood cells; OR, adjusted odds ratio; CI, confidence interval
